# Challenges of the organizational structure of county health network in Iran: findings from a qualitative study

**DOI:** 10.1186/s12913-022-08104-0

**Published:** 2022-05-28

**Authors:** Ahmad Tahmasebi Ghorrabi, Edris Kakemam, Ehsan Moradi-Joo, Nayeb Fadaei Dehcheshmeh

**Affiliations:** 1 Department of Health Services Management, School of Health, Ahvaz Jundishapur University of Medical Sciences, Ahvaz, Iran; 2 Tabriz Health Services Management Research Center, Tabriz University of Medical Sciences, Tabriz, Iran; 3 Department of Management, Policy and Health Economics, School of Management and Medical Informatics, Kerman University of Medical Sciences, Kerman, Iran; 4 Department of Public Health, Shoushtar Faculty of Medical Sciences, Shoushtar, Iran

**Keywords:** Organizational structure challenges, Health network, Healthcare system, Primary healthcare

## Abstract

**Background:**

Primary healthcare with the right structure is the base for any highly efficient healthcare system to achieve better health outcomes at the lowest cost. Challenges of this system, including structural weaknesses, are one of the factors of inefficiency. Therefore, the purpose of this study was to identify challenges of the organizational structure of county health network in Iran.

**Methods:**

An exploratory qualitative face-to-face semi-structured interviews were carried out with 21 key informants including experts and managers in Ahvaz-Iran. Purposive sampling method with maximum diversity were used. Interviews were recorded digitally and transcribed verbatim. Interview transcripts were analyzed based on a thematic analysis approach via NVivo-11.

**Results:**

In analysis of the interviews, after removing the duplicate codes and merging similar items, finally 6 main challenges and 56 sub-themes were obtained. The themes of structural challenges included formalization, complexity, centralization, culture, environment, and resources.

**Conclusions:**

Based on the present situation, the challenges in the current organizational structure and a change in the goals and strategies of the healthcare system in Iran, the appropriate structure needs to be designed and implemented at different levels in accordance with the goals and strategies. The separation and independence of health centers management and hospitals (treatment) in the county can provide a basis for understanding the challenges to the provision of health services.

## Background

An appropriate organizational structure plays a major role in the efficiency and improvement of human resource performance in any organization [1]. Also, well-designed organizational structure leads to effective implementing of strategies, achieving organizational goals, identifying roles and functions of different work units, reducing duplication, facilitating communication and relationships within the organization and increasing efficiency in providing services [2]. In this regard, the healthcare system also includes organizations, institutions, groups and individuals in public and private sectors involved in policy-making, resource production, financing and providing individual and public health services with the aim of recovering, promoting, and maintaining people health. The main goals of the healthcare system include providing, maintaining and promoting the health of people of the society, responding to people expectations and financially supporting them [3]. Also, intermediary goals of access, safety, quality, efficiency, justice and resilience also considered as prerequisite for the primary goals in this system. The common point of all healthcare systems is that people are the main recipients of health services and consider right to choose for themselves [4]. The most important historical event in the development and transformation of health services is the decision of international community to accept primary health care (PHC) to achieve community justice in access to primary health services [5]. PHC includes principles and strategies for organizing healthcare systems [6]. Likewise, PHC with the right structure is the base for any highly efficient healthcare system to achieve better health outcomes at the lowest cost [7]. Studies have revealed that the demand for PHC services has increased in recent years due to changes such as increasing complex chronic diseases, increasing elderly populations along with numerous complications, lack of manpower, geographical dispersion, increasing health costs and development of new technologies [8].

The study conducted by Nekouei Moghaddam (2012) refereed to various achievements of the PHC system in Iran and showed weaknesses and challenges of this system, including structural weaknesses, despite its great achievements [9]. The study conducted by Zanghaneh Beigi (2016) attributed the main causes of the inefficiency of the healthcare system structure in Iran to change in prance of diseases, change in the pattern of demand and change in the main causes of death [10]. Based on the World Bank’s report, problems of Iran's healthcare system the structure area include a strong focus on decision-making, the multiplicity of service provision systems, and the excessive dispersion of this system, especially in cities. Finally, the current organizational structure of PHC in Iran is highly inefficient [11]. Various studies have also indicated that the trend of fundamental changes in the area of health in Iran, such as the implementation of family physician plan, referral system and health revolution plan, reflect changes in the goals and strategies of Ministry of Health and Medical Education (MOHME) at macro level. For this purpose, the first and most basic step after passing or changing the goals and macro strategies of an organization and converging the general policies of the healthcare system is to change the structure, attitudes and policies of the use of essential resources [10, 11]. The need for flexibility to adapt to a changing world is an unavoidable necessity, and selecting an appropriate organizational structure is one of the prerequisites for successful adaptation to changes.

Although the most important function of the structure is to achieve organizational goals, its dimensions (complexity, formalization and centralization) indicating the internal characteristics of an organization are crucial and the intensity or weakness of each is effective in shaping the general structure of organization [12]. Hence, many countries are restructuring the structure of their ministries to make them more effective for fundamental reforms in the area of health and to have a positive impact on achieving the goals of national health. For this purpose, creating organizational structures that are in line with the goals and strategies of national health policies is an essential [2]. The present study was conducted with the aim of investigating and identifying the challenges of the organizational structure of health network of county affiliated to Ahvaz Jundishapur University of Medical Sciences (AJUMS) and providing solutions to eliminate structural defects and correct them and create a suitable environment for efficient implementation of health programs, given the change in the goals and strategies of the healthcare system in Iran, transformations in the health needs of the society, change in the appearance of diseases and new issues and the extensive changes in various social, economic and political areas.

## Methods

### Study design and setting

In current study, an exploratory qualitative approach with semi-structured interview of participants, was undertaken in 2019. The qualitative approach was chosen as it extracts rich data and detailed insights of the phenomenon. The MOHME is responsible for all aspects of planning, leadership, supervision, and evaluation of health services in the country, including training of human resources for health at all levels. Moreover, at regional level, Medical Sciences universities were founded. Universities function independently but under the general rules and policies of MOHME. In addition, health networks at local level are the most natural administrative level promoted by WHO for health delivery. The networks comprise City Health Centers and City hospitals. Also, each city health center consists of urban and rural Comprehensive Health Service Centers (CHSCs). Primary health care in urban centers is provided by the urban CHSCs and its affiliated health bases, and the same services in rural areas are provided by the rural CHSCs and its affiliated health posts. This study was conducted in the health networks of the county affiliated to AJUMS. Ahvaz is a city in the southwest of Iran and the capital of Khuzestan province. Ahvaz's population is about 1,300,000. It should be noted that the structure of the network system in Ahvaz is a similar example of the structure of the health system in Iran.

### Recruitment of participants

The participants of the study included key informants (Faculty member), experts (in health, medical, and human resources areas), and managers (Top-level, Middle-level and Operational) working at AJUMS and health networks of county. In addition to having knowledge on the network system and its structure in the county, they had adequate experience at different levels of healthcare system (at least 5 years of employment history). In the present study, purposeful sampling method was used. This method is used when the researcher intends that the views, opinions and attitudes of certain people to be available in the resulting information [13]. In the present study, maximum diversity was maintained in selected samples and those individuals were included into the study who had different and inconsistent views and opinions on the subject of study [13].

Also, since the sample size is not accurate criterion in qualitative studies, sampling continued until data saturation was achieved. Data saturation is often a guide to deciding how many interviews are enough. In this method, the sampling will end when no new information is added during data collection and the researcher faces information that merely confirms the previous points during data collection stage [14]. Finally, based on the data saturation principle and purposeful sampling method with maximum diversity, the participants of this study included 21 managers and experts of different levels of the university, including the health network of the county, deputies and faculty members. In addition, all of the interviews had been done by EM and ATG.

### Data collection

For data collection, semi-structured depth interviews were conducted. After obtaining informed and written consent from the participants and explained the objectives of the study, face to face interviews were done in agreed time in participants' workplace (EM, ATG). The interviews were recorded by a digital recorder. They took between 30 to 45 minutes and data were collected between August and October 2019.

We designed the interview questions based on the objectives of the study and the opinion of the experts. The validity of the interview guide was evaluated by conducting a pilot study (three individual interviews) and the necessary corrections were made to the guide. Memos helped create next interview questions. The sessions started with simpler questions and interviewees were given less time to think about the answers. Subsequently, more fundamental and challenging questions were posed:

“What’s your opinion about the current function of the organizational structure of the health network of county in Iran?”

“What are the challenges of the organizational structure of the health network of county in Iran?”

“What are the solutions for improving the organizational structure of the health network of county in Iran?”

Probing was conducted according to reflection of each participant on organizational structure, such as perception of the organizational structure, services in the health network of county, and the challenges and facilitators to providing health services in Iran. At the end of the interviews, the final questions were summarized. Coding was done simultaneously by two researchers, and then the codes were compared and agreed upon.

### Data analysis

We used NVivo Software (version-11) to manage the data and facilitate analysis. The transcribed interviews were analyzed using thematic analysis approach. In this approach, important components of the message content and the identified data are taken into consideration and sometimes are compared with other similar figures in whole population [15]. This process is the systematic classification of data through which codes and themes emerge [16]. This method enables researchers to use a regular method to examine large amounts of data in research and to categorize the results [17]. Based on this method, analysis and interpretation were performed in 5 stages, including the researcher familiarity with the data, generation of initial code for the concepts, identifying the themes, the reviewing of the themes and numbering of the themes.

The recorded interviews were transcribed verbatim and they were verified by the interviewer before analysis begins. The transcribed data were read and reviewed multiple times individually by the study authors. Then, meaning units were identified and initial codes were extracted. In the following, codes are integrated and then, classified according to similarities. Finally, the concept and hidden content of data were taken out. Two researchers (NFD, EK) reviewed and coded of the transcripts independently and in duplicate using open coding.

The concepts obtained from the interviews were organized into codes which were summarized and categorized to extract the themes. The main themes and sub-themes were categorized and evaluated. In order to ensure the validity and reliability of the data, the participants were asked to verify the data and the codes extracted. Codes that did not express their views were corrected. Finally, the final report of theme extraction was sent to a group of experts to receive their comments about the classification. Finally, by comparing the observed relationships, concepts, contradictions, and theories, the intended themes were extracted from the findings.

The credibility criterion was achieved by prolonged engagement and member checking. Peer debriefing and external checking ensured performance confirmability and reliability. Two observers reviewed all transcripts, codes, and categories. Lastly, this process concluded with several discussions among the research team on areas of disagreement before achieving final consensus. Considering maximum variation during sampling, we tried to enhance transferability.

## Results

### Demographic characteristics of the participants

Twenty-one people participated in this study. The mean age of the participants in this study was 48.1 years and their mean work experience was 15.9 years (See Table 1).

**Table 1:** Demographic information of the interviewees (*n*=21)

Variables	Frequency	Percent
**Sex**		
*Male*	17	81.0%
*Female*	4	19.0%
**Education**		
*Medical Doctor (M.D)*	12	57.1%
*PhD in health services management*	5	23.8%
*PhD in other field*	4	19.1%
**Position**		
*Top-level manager*	5	23.8%
*Middle-level manager*	4	19.0%
*Operational manager*	2	9.5%
*Faculty member*	4	19.0%
*Expert*	6	28.7%

#### Challenges of the organizational structure of the health network

After reviewing the views and opinions of the participants in the study, six main themes were evident in the perspectives of the interviewees. Themes included ‘formalization', 'complexity', 'centralization', 'culture', 'environment', and 'resources'. (See Table 2).

**Table 2:** Challenges of the county health care network

Theme	Sub-themes	Frequency
Complexity	Administrative and financial duplication in the network headquarters, health center and hospital	16
	Multiplicity and excessive expansion of organizational levels and scope of vertical and horizontal monitoring (lack of agility)	19
	High distribution of network subsidiaries geographically	17
	Extensive scope of monitoring	18
	Late understanding of health and hospitals problems by network	10
	Multiplicity and diversity in clients and customers	12
	Lack of coordination and interference in the duties of subordinate units (weakness in coordination)	20
	Multiplicity of programs, processes, increased responsibilities and high volume of activities	20
	Complexity and difference in the nature of services and types of activities (health, treatment, food and medicine, pre-hospital, etc.)	16
	Incongruity between manpower and duties (health, treatment, food and medicine, pre-hospital, etc.)	11
	Multiplicity in responsible and accountable centers (multiple levels of management) (network manager, head of health center, treatment deputy, head of hospital and head of medical emergencies)	12
	Conflict of interest in network policies and conflict between different departments (network headquarters, health and treatment) /lack of integration and internal cohesion	14
	High dynamics in the field of health and the need for a fast response to challenges and problems	14
Formalization	Uncertainty in the scope of authority and responsibility in practice	19
	Excessive administrative bureaucracy	20
	Ignoring guidelines, rules and regulations	18
	lack of belief in the administrative structure and hierarchy	17
	Dismissal and appointment of managers based on individual decisions	11
	person-centered management	17
	Lack of clear organization and structure for deputies (except for health)	18
	Lack of transparency in the division of managerial duties among the network system components	15
	Weaknesses and defect in the control and monitoring system	19
	lack of paying attention to the organizational structure in practice, lack of observing the hierarchy and reducing the scope of responsibility and accountability of operational managers	19
	improper division of work	12
	Changes in service packages and job descriptions	18
Centralization	Focus of power (financial and administrative and decision-making powers) in the network manager and headquarters	18
	Inadequate authority of managers in hospitals and health centers	19
	Relocation of resources (financial, manpower, equipment) by the manager and the network headquarters in different departments (network headquarters, hospital, health center and emergency)	19
	High level of responsibility and high work pressure on the network manager in the county	18
	inefficiency in making decisions due to structural barriers	14
	Lack of application of communication and information system at decision-making levels	17
	Weakness in communication (between network headquarters and hospital / between network headquarters and health center and affiliated units)	18
Environment	Continuous political, economic, social and environmental changes	10
	Change in national divisions	12
	Changes in the appearance of death and disease	9
	Epidemiological transition	10
	Nutritional transition	10
	Demographic transition (demographic change)	12
	Not considering the social determinants of health in the structure of the network system	15
	Ignoring the role and position of the private, public, and charity sectors in the organizational structure	11
	Ignoring the role and position of the people (citizen-oriented and responding to needs of the people)	9
	Imposing the duties of other departments on the network system	5
	Lack of proper organization in coping with environmental threats	11
Culture	The effect of organizational culture on the structure of network system	14
	Ignoring local (regional) culture in the structure of network system	14
	Increasing and changing people expectations	18
	Increasing and changing expectations of employees	12
	non-importance of competence and seniority in organizational promotion and managerial positions	13
	Instability of management in the network, hospital and health center	16
Resources	Inconsistency between resources and the structure of the network	16
	Lack of financial resources and funds to achieve objectives	15
	Lack of manpower	15
	Lack of equipment	15
	Inconsistency between line and headquarters staff	13
	Lack of necessary consistency in the composition and distribution of organizational positions of units	14
	Unfair distribution of resources	16

##### Theme 1. Complexity

Complexity refers to level of separation that is in an organization. It is measured through three dimensions: horizontal, vertical, and spatial [18]. One of the structural challenges reported by most interviewees was the complexity of the current organizational structure. In this area, 13 sub-themes were obtained from interviews with experts. The multiplicity and excessive expansion of organizational levels and the scope of vertical and longitudinal monitoring (lack of agility), lack of coordination and interference in duties of subunits (weakness in coordination), multiplicity of programs, processes, increasing responsibilities and high volume of activities are major causes of complexity in the current structure. Based on the interviewees, the complexity of the current structure prevents the dynamism of the structure, slows down organizational communication, and causes poor coordination.

“The structure of the health network in cities is very wide in terms of organizational levels, geographical distribution and variety of tasks and the structure does not have the necessary agility.” [Interviewee 9]

“*There is a parallel operation in administrative and financial affairs in the health network headquarters, the county health center and the county hospital*.” [Interviewee 13]

##### Theme 2. Formalization

Formalization is an important aspect of organizational structure that refers to the need and application of regulations, procedures, guidelines, and job descriptions in the organization, and defines the framework of employees’ duties and authority [19]. This challenge was more important than the other two structural challenges and had the maximum consensus in all cases mentioned by the interviewees. In this area, 12 sub- themes were identified. The most frequent and emphasis in this area included uncertainty in scope of authority and responsibilities in practice, redundant bureaucracy and high administrative hierarchy, weaknesses in the control and monitoring system, lack of attention to organizational structure in practice, lack of observing hierarchy and reduced scope of responsibilities and accountability of managers. According to the participants, the formality in the current structure has improper planning.

“*Unfortunately, network administrators do not pay attention to organizational structure in practice and not paying attention to the hierarchy reduces the limits of responsibility and accountability of operational managers*.” [Interviewee 15]

“*Management in the health network system is more person-centered and personalized, and after change each manager, a lot of changes are made in the processes*.” [Interviewee 20]

##### Theme 3. Centralization

This challenge is one of the most controversial components of organizational structure. It refers to power of decision-making in the organization [20]. Based on the participants of this study, this structure focuses on high power and decision-making at high organizational levels, which weakens communication at organizational levels and between different units and slows down decision-making. In this area, 7 sub- themes were identified, which the most frequent of them were insufficient scope of authority of managers in hospitals and health centers and replacement of resources (financial, human resources, equipment) by the manager and network headquarters in different departments (network headquarters, hospital, health center and emergency).

“Financial and administrative power and decision-making powers are concentrated at the level of the health network headquarters, and the powers of managers in hospitals and health centers are insufficient.” (Interviewee 6)

“*Financial resources, manpower and equipment of different departments (hospital, health center and emergencies) are transferred by the network manager and staff without coordination with the health center and hospital.”* (Interviewee 11)

##### Theme 4. Environment

One of the factors influencing the organizational structure is the factors outside the organization, such as institutions and forces that affect the performance of the organization. Organizations also have little or no control over the environment, and this issue highlights the importance of a proper structure to environmental change [21]. According to the participants, the current organizational structure of the healthcare network of the cities is inappropriate for the changes around them. The most important factors included change in the appearance of death and disease, epidemiological transition, nutritional transition, demographic transition (demographic change). In this area, 11 sub- themes were identified, and not considering the social determinants of health in the structure of the network system from the perspective of the interviewees was the most important challenge in this area.

“*Environmental changes (political, economic, social and environmental) affect the tasks and responsibilities of the health network*.” (Interviewee 9)

“*Social determinants of health are not included in the structure of the network system*.” (Interviewee 16)

##### Theme 5. Culture

Organizational culture, or the values governing the organization, is one of the factors influencing the organizational structure. Also, the organization and its structure should be aware of the cultural issues of the society [22]. In this area, 6 sub- themes were identified, and from the perspective of interviewees, increasing and changing people expectations was the most important issue in this area. From the perspective of participants in the interview, the current organizational structure is indifferent to the expectations of people, local culture and staff, leading to reduced people involvement in health issues and reduced staff motivation.

In this regard, one of the participants mentioned “*Over the years, the expectations of people and staff have changed a lot, so it is necessary to adapt the structure of its network system to these changes.”* (Interviewee 10)

“*Competence and seniority system has no place in organizational promotion and management positions in health care networks*.” (Interviewee 19)

##### Theme 6. Resources

Resources (financial, human, equipment, physical, etc.) are important factors in the survival of the organization and achieving the goals [23]. This area was the most challenging and important issue among the challenges from the perspective of interviewees. In this area, 7 sub- themes were identified. Based on the interviewees, due to the extensive structure of the network system, coverage of services and the large number of units providing health services, lack of human and financial resources and equipment are significant. Also, given the defined programs and duties, there is not enough credit for the health network, and if resources are allocated, its distribution will be unfair and more allocated to treatment area and hospitals.

Interviewee 3 stated that "*The health network is facing a lack of resources including financial, human, equipment, physical to achieve its goals and tasks*.”

Also, one of the interviewees said that “*Distribution of resources in the health sector is not equality.”* (Interviewee 14)

## Discussion

To make fundamental reforms in the field of health and achieve its goals, it seems necessary to create organizational structures in line with national health goals, strategies and policies given that flexibility is essential to adapt to a changing world [2, 12]. Therefore, the present study was conducted with the aim of investigating the challenges and facilitators of the organizational structure of the health network of county affiliated to AJUMS. The results of the present study revealed that the organizational structure of the health network of county face many challenges. Various studies have also reported problems in the current organizational structure, especially at the environmental level (county health center and urban and rural health centers) [10]. In this regard, the multiplicity and excessive expansion of organizational levels and the scope of vertical and horizontal monitoring (lack of agility), non-coordination and interference in the functions of subordinate units (weakness in coordination), multiplicity of programs, processes, increasing responsibilities and high volume of activities were among the most important cases of complexity in the current structure. In a prior study conducted in Iran have been demonstrated that high complexity of the system due to the existence of multiple units and the creation of new units, and it reduced the efficiency of the system in achieving its basic goals [10]. A study has been done by by Nekuei Moghaddam showed that improper performance, providing parallel services and lack of intra-sectoral and inter-sectoral coordination are among the problems that will ultimately lead to reduced people satisfaction with the healthcare system [9]. Elimination of network headquarters and prevention of overlap, parallel work and duplication of some headquarter units such as service, transportation, administrative affairs, financial affairs and procurement (reducing the number of organizational levels and management layers in network system) seem to be necessary.

In the present study, according to the participants, the high formalization of current structure led to lack of proper planning. Studies have indicated that the formalization of the system is very high due to many programs and guidelines and reduces creative power and motivation of manpower. The volume of these programs has increased since the establishment of health networks, but none of the unnecessary programs has been removed so far [9, 10, 24]. One of the most controversial and important challenges of the current structure in the present study is the high centralization of power and decision-making at high organizational levels, which has led to poor communication at organizational levels and among different units and slow decision making. Several studies have also revealed more attention needs to pay to centralization dimension than other dimensions and it has been considered as one of the main disadvantages of the existing system. Also, all studies conducted in this area have reported satisfactory results from decentralization [9, 10, 25, 26]. The World Bank has also reported that the current structure is inefficient due to the multiplicity of centers and high focus and needs to be reformed [24]. Administrative and financial independence (delegation of authority) of health center and hospital in the county can decrease these challenges. In the present study, the current structure had content challenges at environment, culture and resources dimensions. According to participants' perspective, the most important of them included inappropriateness of the current structure to surrounding changes, indifference of the current structure to people expectations, local culture and staffs, and unfair distribution of resources. Considering the challenges related to the environment and culture, various studies have shown the current organizational structure faces the challenges of lack of sense of ownership among members of society to healthcare system, low participation of society in solving health problems, lack of flexibility and accountability in the healthcare system, insufficient training of health staffs on communication and counseling skills [27–31]. Reengineering and organizational restructuring of the health network in county are essential to needs of society, health problems, mission, and goals of the healthcare system. Studies have also indicated that the current structure in the area of resources faces the challenges of parallel systems of financing in the system, lack of coordination in financing, lack of separation in financing and providing services, inadequacy and lack of financial resources, unfair distribution of resources and lack of coordination between funding sources and service packages needed [5, 32, 33]. The results of several studies are in line with the results of the present study on content challenges. Separation and independence of health center management and hospitals (treatment) in the county (elimination of network management layer and creation of a structure consisting of two separate operational managements in the county (health and treatment management) seem to be essential. In the content dimension, the current situation of the healthcare system in Iran lacks a proper information system. New technologies such as automation and the Internet also have many problems and differences in different universities of Iran and serious attention should be paid to these areas [34]. The present study also emphasized the importance of appropriate technology and information systems in facilitating and improving the current structure. Paying attention to the content dimensions influencing the healthcare system in the current situation, especially the environmental and cultural dimension of the organization, can help the system in achieving its main goals, since content dimensions represent the whole organization and affect the structural dimensions [10]. Despite the challenges in the current structure, interviewees of this study stated that this structure has facilitators such as service coverage and network expansion (network expansion to the most environmental level), leveling of services, diversity of human resources in terms of different specialties from lowest to highest level, program-based and indicator-based structure and having technically and procedurally codified programs, protocols, and guidelines. Several studies have also reported that the current structure has strengths such as network expansion, referral system, full access, community health workers (Behvarz), provision of services in villages, close communication with society, appropriate and comprehensive regulations and guidelines in the field of clinical and health [5, 35–37].

In the current situation, due to the challenges related to the current organizational structure, the need to create new structures in the healthcare system due to changes in various areas, the need to reform the organizational structure, re-engineering the current structure and revising the information management system have been proposed in various studies [10, 34, 36]. Changes in the healthcare system can also be seen in other countries. For examples, we can refer to changes in the healthcare system of some European countries [35, 37]. The study conducted by Tabrizi et al. also showed that considering the social and economic changes, the current structure of the system should be reformed to be tailored to phenomenon of chronic diseases, incidents, and population aging [5]. In the present study, the interviewees emphasized on reengineering and restructuring based on the needs of the society, health problems, mission, and goals of the healthcare system. Thus, it is recommended to strengthen and maintain the existing strengths in the current structure and to pay attention to the facilitating factors inside and outside the organization and use the solutions presented in this study to make desired reforms. Finally, the appropriate structure needs to be designed and implemented at different levels in accordance with the goals and strategies. In this case, the organizational structure of network management in the city will provide different services to people with more capacity and speed, otherwise, the healthcare system will face many problems in providing health services, leading to reduced quality and people dissatisfaction with the healthcare system.

### Strengths and Limitations

The finding of this study showed that separation and independence of health center management and hospitals (treatment) in the county (elimination of network management layer and creation of a structure consisting of two separate operational managements in the county (health and treatment management) can provide a basis for understanding the challenges to the provision of health services. Conflict of interests of managers and employees in expressing challenges and solutions was the limitation of this study.

## Conclusion

The current structure has several strengths and achievements, especially in the field of health, but given the current situation, the challenges in the current organizational structure and changing the goals and strategies of the healthcare system in Iran, reengineering and organizational restructuring of the health network in county are essential to needs of society, health problems, mission, and goals of the healthcare system. The current structure had content challenges at ‘formalization', 'complexity', 'centralization', 'culture', 'environment', and 'resources' dimensions. The organizational structure of network management in the city will provide different services to people with more capacity and speed, otherwise, the healthcare system will face many problems in providing health services, leading to reduced quality and people dissatisfaction with the healthcare system. The appropriate structure needs to be designed and implemented at different levels in accordance with the goals and strategies. The separation and independence of health center management and hospitals (treatment) in the county can provide a basis for understanding the challenges to the provision of health services. It is recommended that future studies be conducted in the field of reengineering and restructuring of universities of medical sciences and health networks.

## Data Availability

The datasets used and/or analyzed during the current study are available from the corresponding author on reasonable request.

## Abbreviations

PHC: Primary health care
MOHME: Ministry of Health and Medical Education
AJUMS: Ahvaz Jundishapur University of Medical Sciences
CHSCs: Comprehensive Health Service Centers
